# Parent Training Interventions for Toddlers with Autism Spectrum Disorder

**DOI:** 10.1155/2014/839890

**Published:** 2014-05-07

**Authors:** Audrée Jeanne Beaudoin, Guillaume Sébire, Mélanie Couture

**Affiliations:** Université de Sherbrooke, Sherbrooke, QC, Canada J1H 5N4

## Abstract

*Background*. Now that early identification of toddlers with autism spectrum disorder (ASD) is possible, efforts are being made to develop interventions for children under three years of age. Most studies on early intervention have focused on intensive and individual interventions. However, parent training interventions that help parents interact and communicate with their toddlers with ASD might be a good alternative to promote the development of their child's sociocommunicative skills. *Objective*. This review aims to systematically examine (1) the use of parent training interventions for children with ASD under three years of age and (2) their effects on children's development, parents' well-being and parent-child interactions. *Methods*. Systematic searches were conducted to retrieve studies in which at least one parent was trained to implement ASD-specific techniques with their toddlers (0–36 months old) with a diagnosis of or suspected ASD. *Results*. Fifteen studies, involving 484 children (mean age: 23.26 months), were included in this review. Only two of them met criteria for conclusive evidence. Results show that parents were able to implement newly learned strategies and were generally very satisfied with parent training programs. However, findings pertaining to the children's communication and socioemotional skills, parent-child interactions, and parental well-being were inconclusive.

## 1. Introduction

Autism spectrum disorder (ASD) is characterized by difficulties in social interaction and communication and by repetitive and stereotyped behaviors. With an estimated prevalence of up to 1/88 children in the US [1], ASD is amongst the most frequent and severe developmental disorders. In the United States, ASD is associated with an estimated annual cost between 35 and 90 billion dollars [2, 3].

In response to parents' concerns about their child's development that are present as early as 12 months of age [4], knowledge about and strategies to identify young toddlers with ASD are emerging [5]. It is now possible to identify a child with ASD in the first two years of life based on sociocommunicative behaviors [6].

Now that early identification of toddlers with ASD is possible, efforts must be made to develop interventions for these young children. In 1987 and 1993, Lovaas and colleagues first described the potential efficacy of early intervention for children with ASD by reporting less restrictive school placement and higher IQ in the group of children who received an intensive behavioral intervention compared to children in the control group [7, 8]. More recently, early interventions have been associated with greater developmental gains and more reduction in autistic symptoms than interventions provided later on [9, 10]. Reilly and colleagues [11] reported that typically developing children between 12 and 24 months of age go through an important period of development that results in more advanced social communication abilities. Interventions in this time frame are crucial to prevent an increase in the gap between children with ASD and their typically developing peers [12]. Also, the significant neuroplasticity in young children emphasizes the importance of very early intervention [13]. In fact, many clinical studies [14–20] have described the interaction between infants' brains and their social environment and its impact on the development of infants' social and language brain circuitry [12]. Knowing that each experience influences the brain structure and function of young children, interventions with toddlers with ASD have the potential to influence their developmental trajectories towards a more typical and behavioral development [12, 13, 21].

Most research on early intervention focused on early intensive and individual interventions (20 to 40 hours per week). Despite their demonstrated efficacy for preschoolers with ASD [8, 22], intensive individual interventions are expensive and time-consuming and were originally developed for children between three and five years of age who have different learning needs than younger children. Infants from 12 to 36 months of age have specific developmental abilities and learning capacities, which require interventions that are tailored to them. Parent training interventions may be an effective alternative to support the learning capacities of toddlers with ASD [23]. Moreover, parent training interventions are based on language development in very young children, which has been demonstrated to be dependent on the language used by their parents [24]. Thus, teaching parents how to interact and communicate with their toddlers with ASD through parent training interventions might promote the development of their child's sociocommunicative skills.

Few researchers have reviewed the effect of parent training interventions specifically for young children with ASD. At this time, most of the literature on early parent training interventions for children with ASD focuses on children under seven [25–27] or nine years of age [28]. No review focusing on parent training interventions for toddlers under 3 years of age has been published to date. Given the previously described potential benefits of very early parent-mediated interventions, there is a need to understand if this type of intervention is effective specifically for those young children. This review aimed to systematically examine the use of parent training interventions for children from 0 to 36 months of age at risk of or with a diagnosis of ASD and their effects on children and parents. In particular, the following questions were addressed.

1. What are the parent training programs used for children from 0 to 36 months of age with a diagnosis of or suspected ASD?
2. What are the effects of parent training programs on (a) child development, (b) parental well-being, and (c) parent-child interactions?

## 2. Method

### 2.1. Search Procedures

Systematic searches were conducted in May and June 2013 in four electronic databases: CINAHL, ERIC, PubMed/Medline, and PsycINFO. The search was limited to articles published in English or French in peer-reviewed journals. Searches were conducted using combinations of the three keywords (and synonyms): “autism spectrum disorder” (e.g., autism, pervasive developmental disorder), “toddlers” (e.g., infants, preschoolers, and early intervention), and “parent training” (e.g., parent coaching, parent-mediated intervention, and caregiver-mediated intervention). We also searched in the Cochrane Database of Systematic Reviews, looked in Google Scholar, and examined bibliographies of systematic and nonsystematic reviews found in any of the six databases. Reference lists of articles that met inclusion criteria were also examined to identify articles that had not been found through electronic searches. We found eleven additional studies using these alternative procedures.

### 2.2. Inclusion and Exclusion Criteria

To be included in the review, studies had to meet four inclusion criteria as follows:

1. all children had to be at risk of or have had a diagnosis of ASD (including pervasive developmental disorder, autistic disorder, Asperger syndrome, and pervasive developmental disorder not otherwise specified);
2. more than 50% of the children had to be 36 months of age or less at the time of recruitment (if this information was not available, the mean age of participants at recruitment had to be under 36 months) and all children in the study had to be 5 years old or less;
3. at least one parent (referring to fathers, mothers, foster parents, grandparents, or other relatives) had to be trained by an education or health professional to use ASD-specific techniques to promote the child's development. Parents must have received ongoing supervision and support from the professional. The training could involve individual and/or group coaching at home and/or in a clinic environment. Studies that assessed different types of interventions (e.g., individual intensive interventions) were excluded from the review if they did not report effects of parent training interventions separately from other types of interventions;
4. effects of the intervention had to be reported by quantitative data. Quantitative data from mixed methods studies could be included if they reported intervention effects.

### 2.3. Data Extraction and Coding

Studies included in this review were summarized in terms of (a) participant characteristics, (b) dependent variables and measurement tools, (c) intervention characteristics (including program type, strategies, setting, frequency, duration, and goals), (d) intervention outcomes (directly after intervention and at followup, when appropriate) on children, parents, and parent-child interactions, and (e) certainty of the evidence.

Parent training outcomes (effects on children, parents, and parent-child interactions) were summarized as reported in the original paper (i.e., pre-post change, statistical significance, or effect size). Considering that a systematic review is a scientific tool that can be used by healthcare providers, consumers, researchers, and policy makers [29] to “evaluate existing or new technologies and practices efficiently and consider the total available data” [30], outcomes in each article were then classified into four categories (positive, mixed, no, and negative effects) according to the magnitude of changes due to parent training interventions. Previous systematic reviews used the three first categories [31–33]. Results were classified as “positive” if visual analysis of the results of single case studies showed an increase in abilities for all participants between baseline and intervention or if there was a statistically significant between-group difference in experimental designs. “Mixed” results were defined as improvement in some but not all participants in single-case designs or a trend short of statistical significance in between-group designs. If no participants in single-case studies benefited from the intervention or if there was no statistically significant improvement in between-group designs, results were classified as “no effects.” Finally, the research team added a fourth category to the original 3-level classification, namely, “negative” effects. Results were classified as “negative” if visual analysis of the results of single-case studies demonstrated deterioration in abilities in most participants between baseline and intervention or if there was a statistically significant between-group difference in favor of the control group in experimental designs. However, none of the studies reviewed reported negative effects of the intervention.

The studies' methodological rigor could be assessed based on several classification systems. The research team used a 3-level classification system (suggestive, preponderant, and conclusive evidence) used in previous reviews [31, 32] to summarize the level of evidence for each study reviewed. “Suggestive” evidence was the lowest level of evidence. Studies classified at this level did not use a true experimental design. They might have used pre- or quasi-experimental designs including an AB-design or pre-post intervention design without a control group. The next level of certainty was “preponderant” evidence, which means that there was a strong, but not irrefutable, conclusion. Studies included in this second level of certainty needed to (a) use a true experimental design, (b) have adequate interobserver agreement (i.e., at least 80% fidelity) for at least 20% of evaluation sessions, (c) have an operationally defined dependent variable, and (d) provide sufficient details about the intervention for replication of procedures. The strongest form of evidence was “conclusive.” To provide conclusive evidence, studies had to comply with all the attributes of the preponderant level plus contain (a) design features that provided at least some control for alternative explanations for intervention outcomes and (b) a measure of treatment fidelity to assess the degree of implementation of treatment-specific strategies by parents and/or therapists throughout the program.

## 3. Results

### 3.1. Description of Studies

Six hundred sixty-nine (669) articles were retrieved from the electronic searches. After removing duplicates and elimination based on the title, 174 abstracts remained. Based on these 174 abstracts, we eliminated 86 articles. The remaining 91 full-text articles were further assessed for eligibility. At this point, 75 articles were excluded because they described intervention models rather than assessing their effects (*n* = 7), children had developmental disabilities other than ASD (*n* = 4), children were older than 36 months (*n* = 60), studies reported exclusively qualitative data (*n* = 1), studies did not report results specifically for the parent training intervention (*n* = 1), or studies focused exclusively on a specific component of parent training rather than the whole intervention (*n* = 2). Of the remaining 16 articles, Vismara et al.'s paper [34] concerned the single participant included in a previous case study [35]. Therefore, we excluded the single case study [35] to avoid redundancy. The research team also contacted Gerald Mahoney, who ascertained that most children in Mahoney and Perales' studies [36, 37] were different. Despite a little overlap, these two studies were included in the review. A total of 15 studies were analyzed for this review.  Table 1 describes the main features of each of these 15 articles.

#### 3.1.1. Participants

A total of 484 children with a mean age of 23.26 months were included in this review. Those children were diagnosed with ASD (*n* = 248; [34, 36–40, 42–45]) or identified as being at risk of ASD based on either the presence of early markers (*n* = 156; [10, 41, 46]) or because they were infant siblings of probands with ASD (*n* = 80; [47, 48]).

Two hundred seventy-seven (57%) parent-child dyads received a parent training intervention, whereas the remaining 207 children were controls. Gender was reported for 253 of the 277 toddlers involved [10, 36, 37, 39–45, 47]; 192 children were male (76%) and 61 were female (24%).

Primary caregiver gender was detailed in seven of the 15 studies reviewed [36, 37, 40–42, 45, 47]. Most primary caregivers who participated in parent training programs in these studies were female (*n* = 95; 99%), compared to only one male primary caregiver (1%). Two papers reported training of relatives other than mothers or fathers. Kasari and colleagues [43] included a grandparent as the primary caregiver for two children. Also, Vismara et al. [34] included a grandmother as an observer during parent training sessions rather than as an active participant, who was the child's mother in this case. The mean age of parents (or other caregivers) receiving training ranged from 27.3 [41] to 36 years [40].

#### 3.1.2. Intervention Types

Parent training interventions for children with a diagnosis of or suspected ASD may be classified based on their theoretical paradigms or their targeted goals.

Based on their theoretical paradigms, interventions may be positioned on a continuum from pure behavioral interventions to sociopragmatic interventions [49]. Interventions based on a behavioral paradigm stem from Lovaas [7] and colleagues' work and are characterized by a high level of structure in which a therapist prompts the child's behavior. When the child responds correctly, the therapist reinforces the behavior. At the other end of the continuum, sociopragmatic interventions are represented by the DIR Floortime model [50]. Sociopragmatic interventions are characterized by following the child's lead through spontaneous play activities and using those activities to enhance the child's learning. Eight of the 15 articles reviewed used interventions mainly based on the sociopragmatic paradigm [36–39, 41, 44, 45, 47] and two articles reported more behaviorally based interventions [40, 48]. The remaining five articles incorporated strategies from both paradigms [10, 34, 42, 43, 46]. For example, the Early Start Denver Model parent training program used by Vismara and colleagues [34] and Rogers and colleagues [10] integrates behavioral techniques, such as repetition and the ABC structure of activities with sociopragmatic strategies, including intervention through activities chosen by the child in natural settings [51].

Interventions can also be classified as either ability-focused or comprehensive. Focused interventions are individual instructional strategies used to develop specific abilities in children with ASD [52]. Ten papers used focused interventions [38, 40–48]. Abilities targeted in these interventions included communication [45, 46, 48], joint attention [38, 40–44], and parent-child relationship [47]. On the other hand, comprehensive treatment models were studied in five articles [10, 34, 36, 37, 39]. These interventions use sets of practices with multiple components for a broader developmental impact on core deficits in ASD [53]. They may include development of communication, social interaction, cognition, play, and autonomy. Notwithstanding the type of intervention, development of communication was a main goal of all 15 papers. In fact, difficulties with communication are a core deficit in ASD and frequently the main concern of parents.

Eight of the 15 articles used a comparison group, which allows for a better estimate of the effect of the intervention on children and parent outcomes. Interventions received by the 233 participants in comparison groups varied widely. Ninety-six of these participants (41%) from three studies [39, 45, 47] did not receive any treatment while, in the other five studies, 137 participants (59%) received treatment “as usual” [44, 46] or a mixture of interventions [10, 38, 43].

#### 3.1.3. Setting

All 15 articles reported individual parent sessions, six of which took place in the family home [38, 39, 41, 44, 46, 47], at a clinic in six studies [10, 34, 40, 42, 43, 45], and either at home or in a clinic in three studies [36, 37, 48]. Mahoney and Perales [36, 37] gave families the choice of setting (clinic- or home-based) for the parent training program. Steiner and colleagues [48] varied the setting of the parent training: 8 sessions took place in a clinic and 3 at home. In this paper, clinic-based sessions were focused on teaching new strategies while home-based sessions focused on generalization of the newly acquired techniques at home. Also, two studies used some parent group classes that took place in a clinical setting [39, 46].

#### 3.1.4. Intensity

Total time of interventions received varied between five and 32 hours (mean = 18 hours). Most studies involved parent training sessions for a total time between 10 and 20 hours [10, 34, 40–43, 47, 48] over a period of two to six months [10, 34, 39, 41–43, 46–48]. Intervention programs that offered more than 20 hours of intervention lasted at least 12 months [36–38, 44]. Only one paper assessed the effects of a shorter, but intensive, parent training program. Wong and Kwan [45] studied an intensive 2-week parent training intervention during which parents attended ten 30-minute sessions.

#### 3.1.5. Teaching Strategies

Studies incorporated a range of strategies to teach parents new skills. Some articles reported the use of only one [42, 43, 47] or two [46] teaching strategies. However, most studies used many different teaching strategies to enhance parents' learning; the mean number of strategies described in the articles reviewed was 4.4 (from 1 to 9). Wetherby and Woods' [39] article reported the most varied use of teaching strategies. Unlike every other study, therapists in this study did not use all of the teaching strategies in the intervention program. They rather personalized the teaching strategies used based on therapists' and parents' preferred teaching and learning styles, respectively.

The most commonly used teaching strategy was didactic teaching by the therapist. Only two studies [39, 47] did not report therapists verbally explaining new techniques to caregivers. Rather than using traditional lectures by the trainer, Wetherby and Woods [39] used video clips to introduce new skills to parents. Other popular teaching strategies included demonstration or modeling of intervention techniques by the trainer [10, 34, 38–40, 44, 48], guided practices of new skills by parents with support and/or feedback from the therapist [10, 34, 38–40, 44, 48], written materials given to parents [10, 34, 39–41, 44], discussion between the trainer and parents on activities to develop targeted child skills [36–39, 41, 44], review of previous techniques [10, 34, 36, 37, 41], and parents having to implement new skills in daily living routines [38, 40, 41, 44, 45].

Other teaching strategies used in some studies included discussions about short-term objectives [38, 39, 44], about generalization of newly acquired skills in family daily routines [10, 34, 39, 44] and about family concerns that were not included in the parent training program [39, 41]. To ensure good comprehension by parents, they were encouraged to ask questions [39, 40], to provide good and poor examples of implementing a strategy [40], and to demonstrate their use of the strategy [45] Also, video feedback [46, 47] and fading of prompts [40] were used in recent studies to help parent implement strategies with their children.

### 3.2. Effects of Interventions

Only four of the 15 studies included a follow-up assessment in their study design [34, 40, 41, 46]. Timing of follow-up assessments varied from one [34] to four months [46] after the end of the intervention.  Table 2 summarizes the effects of interventions for both postintervention and follow-up assessments.

#### 3.2.1. Child Outcomes

Thirteen of the 15 articles reported the effects of a parent training program on children's abilities directly at the end of the intervention (postintervention), and three of these 13 papers reported effects on children's development at followup. However, the results of the 13 studies diverged, partly because of the heterogeneity of the intervention programs proposed and the diverse research methodology used.

As reported in Table 2, some gains in general communication outcomes were reported by Wong and Kwan [45]. More precisely, studies reported a significant increase in the number of words understood [39] and expressed [34], frequency of communication [48], imitative behaviors [34], joint attention [39], and eye contact [39, 41] after a parent training intervention.

However, these gains were not consistently reported across the studies reviewed. In fact, five of the six randomized controlled trials reported few [38, 43] or no [10, 44, 46] gains in sociocommunicative outcomes for children with ASD after a parent training intervention. Although Wong and Kwan [45] reported similar mixed results concerning expressive and receptive communication skills, their study was the only randomized controlled trial that reported positive effects on children's social communication. In fact, parents reported statistically better social relationships with people (*P* = 0.007) for their child after the Wong and Kwan's 2-week parent training program that targeted eye contact, gestures, and vocalizations. Also, both studies that met methodological criteria for conclusive evidence reported divergent results. On the one hand, Carter and colleagues [46] reported no main effect of the intervention on changes in child communication outcomes after the intervention (ES < 0.001). On the other hand, Kasari and colleagues [43] reported significant improvements in engagement states (object engagement ES: 1.09; joint engagement ES: 0.87), frequency of joint attention responses (ES: 0.74), and quality of functional play (ES: 0.88), whereas they did not observe significant improvements in frequency of attention initiations and quality of symbolic play following the 8-week parent training intervention.

Effects of interventions at follow-up assessments were similar to postintervention results. Among the three studies that examined children's communication trajectories after the end of the intervention program, one reported no intervention effects [46], one reported mixed results [40], and one reported positive results [34]. Again, if we examine the results of these studies in light of their certainty of evidence, the only randomized controlled trial available [46] did not report any significant effect of the intervention on children's communication four months after the end of the Hanen* More Than Words* parent education program (ES: [−0.19; 0.16]), as opposed to both case studies [34, 41] that reported more positive long-term outcomes.

No major difference was found between parent-reported results and direct observation measures. In fact, proportions of categorical outcomes (no, mixed, and positive effects) are similar between results from caregiver-reported assessments (no effect: 27%; mixed effects: 36%, positive effect: 36%) and direct observation measures (no effect: 27%; mixed effects: 20%; positive effect: 53%). Furthermore, two studies used both types of measures to assess intervention effects on the same variable and they both reported similar results from parent-reported and professional observation data. More precisely, Oosterling and colleagues [44] did not report any significant improvement in expressive communication after their 1-year parent training program or in the parent-reported MacArthur Communication Developmental Inventory [54] nor in the direct observation rating on item A1 (level of nonechoed language) of the Autism Diagnosis Observation Schedule (ADOS; [55]). Also, Wong and Kwan [45] reported significant gains after their 2-week intensive parent training intervention in parent ratings of their child's communication (*P* = 0.010) on the Language subscale of the Ritvo-Freeman Real Life Rating Scale [56] and in blind assessors' ratings on the Communication and Language subscale of the ADOS (*P* = 0.007).

#### 3.2.2. Parent Outcomes

Considering that parents are the main actors in parent-mediated interventions, it is very important to evaluate the effects of parent training interventions on both parent and child behavior [57, 58]. However, in the articles reviewed, parent outcomes received much less attention than their child's sociocommunicative development (see Table 2).

In general, parents of toddlers at risk of ASD who participated in a parent training program reported a high level of satisfaction with the intervention [41, 46, 48]. These parents also tended to have a high level of fidelity of strategy implementation [10, 34, 40, 43, 48] that was maintained four weeks after the end of the intervention [34]. However, Rocha and collaborators [40] reported higher fidelity of strategy implementation when interventions took place in clinics (mean = 92.7%), compared to the family home, which represented a generalization context (86.8%).

The scientific literature strongly supports the notion that parents of children with ASD often experience higher stress than parents with typically developing children or children with other disabilities [59–63]. Therefore, parent training may be an important strategy for reducing parental stress [64]. However, few (*n* = 2) studies examined the effect of parent training on this very important parental variable. In addition, the results available differ from one study to the next. Wong and Kwan [45] reported a significant decrease in parental stress as measured by the total score on the Parental Stress Index- (PSI-) Short Form (*P* = 0.004) after a 2-week intervention comprising a child-therapist therapy targeting eye contact, gestures, and vocalizations and a program teaching parents how to use corresponding techniques at home. Interestingly, no significant decrease was observed in this study when analyzing parental stress separately for the three subscales of the PSI-Short Form (*P*'s from 0.025 to 0.069). In the other study, the social-pragmatic joint attention-focused parent training program evaluated by Drew and colleagues [38], which lasted longer but was less intensive (i.e., once every six weeks) than Wong and Kwan's program, did not significantly decrease parental stress in comparison to the local services control group (*P* > 0.05).

The effects of parent training programs on other parental variables have received very little attention. Based on data available at this time, parent training interventions seem to be well accepted by parents [47] and may facilitate the establishment of a good therapeutic relationship [10]. Parental variables such as parental competence, self-efficacy, and empowerment were not explored in the studies included in this review.

#### 3.2.3. Parent-Child Interactions Outcomes

As reported in Table 2, 10 of the 15 studies reviewed assessed effects of interventions on parent-child interactions. However, no study reported parent-child interactions with a global measure for the parent-child dyad. Instead, analyses of parent-child interactions were based on the engagement of each partner in the interaction separately. Thus, authors of the ten articles assessing parent-child interactions used either one [34, 41–43, 47] or both [36, 37, 40, 44, 46] of the following outcomes: parent's engagement with the child and child's engagement with the parent.

Seven studies assessed the parent's engagement in interactions with their child using different scales that were all based on observing an interaction period lasting a few minutes during which the parent and child played together. Four of these studies reported mixed results of intervention programs on parental engagement. More precisely, the studies reported a moderate increase in the parent's responsivity [36, 37, 46, 47] and positive effect [36, 37] after the intervention. However, no significant change was reported in directiveness [36, 37, 47] and achievement orientation, which refers to the parent's encouragement of the child's achievement and the quality of praise provided to the child [36, 37] after intervention. Both studies that assessed parent engagement with their child at followup reported that gains observed in the intervention period were not maintained [40, 46].

Eight studies analyzed the child's engagement with the parent during play. Mahoney and Perales [36, 37] reported significant improvement in children's social interactive behavior generally with their parents. However, most studies targeted specific components of children's engagement and reported mixed results. For example, children's responsivity to parents' joint attention bids [40, 43] and children initiating joint attention bids in parent-child interactions [34, 36, 37, 40] increased moderately in most studies. However, Kasari and colleagues [43] did not report greater initiations of joint attention in children who received the parent training intervention compared to a waitlist control group. Kasari and colleagues [43] also assessed the effects of interventions on the types of engagement and found that children increased their joint engagement with their parents and reduced their engagement in object-focused play. An increase in attention toward parents was also reported in three other studies [34, 36, 37]. Interestingly, changes in children's engagement were less significant in generalization contexts such as with the therapist instead of the parent [34] or at home instead of at the clinic where intervention sessions were conducted [40].

Finally, only two studies assessed maintenance of children's improved engagement with their parent and they both reported mixed results. More precisely, the increase in children's engagement with their parents was maintained for most, but not all, children [34, 40].

## 4. Discussion

To sum up, 15 studies on parent training interventions for toddlers with ASD were included in this systematic review. There was substantial heterogeneity between these 15 studies, particularly with respect to the intervention programs and assessment measures used. Consequently, the results could not be compiled in a quantitative analysis such as a meta-analysis. Nevertheless, the research team used two classification systems that described the magnitude of change (positive, mixed, no, or negative effects) and the certainty of evidence (suggestive, preponderant, or conclusive evidence) to summarize the meaningfulness of the differences found in each study. However, most studies on parent training interventions included in the review had low levels of certainty of evidence (as a result of methodological limitations) and the magnitudes of changes were hard to judge (few effect size data available). Thus, despite the use of two classification systems, the results must be interpreted with caution because of the substantial variability and methodological limitations that make comparison between studies difficult.

Despite these limitations, most studies in the present review demonstrated that positive changes can be obtained in young children with ASD following a parent training intervention. In fact, the 15 studies reviewed reported substantial but inconsistent gains in this group of children in communication, socioemotional functioning, symptom severity, and play. These results are consistent with the work of McConachie and Diggle [27], who concluded that there is a good reason to think that the training of specific skills for parents may produce positive changes in children under seven years of age diagnosed with ASD. However, the current lack of experimental studies with large sample sizes that take place in the clinical real world (rather than a university-based clinic) makes it impossible to reach firm conclusions regarding the effectiveness of parent training interventions in supporting the development of toddlers with ASD.

Furthermore, parents' attitudes and skills showed great improvement following parental training interventions. In fact, parents who participated in these interventions for toddlers with ASD consistently reported a high degree of satisfaction. Similarly, a qualitative study exploring the experience of parents of children with ASD from 28 to 37 months of age who participated in the* More Than Words* (MTW) parent education program reported that parents viewed the MTW program as a good starting point for learning how to help their child with ASD [65].

In general, parents also showed high rates of fidelity of strategy implementation after the training period. The ability of parents to learn how to implement strategies adapted to their child and their satisfaction with the parent training show the feasibility of using parent-mediated interventions with parents of toddlers at risk of or diagnosed with ASD. These results are consistent with previous reviews of parent-mediated interventions for older children with ASD [66–68]. For instance, a systematic review of parent training interventions that targeted the communication skills of children 10 years of age and younger with ASD [67] reported similar results. Parents included in that review reported high levels of satisfaction and acceptability with the intervention programs. In addition, they were able to implement communication interventions more accurately following training [67]. Finally, Lang and colleagues' review [67] also reported that parents were still able to implement interventions correctly in six of the seven studies that looked at maintenance data [67]. This was also reported in the only study in our review that included follow-up data on implementation [34].

Also, that review reported mixed effects of parent training interventions on parental stress, which is consistent with the inconclusive results reported by Oono and colleagues [25]. However, this contradicts the hypothesis that parental stress decreases in low-intensity treatments while it increases in high-intensity interventions [69, 70]. Thus, parent training interventions may reduce the number of hours of intervention with a therapist. However, parents in the parent training group rated their workload as higher than those assigned to the intensive early intervention implemented by a therapist [71]. Considering that the workload perceived by parents may be an important factor related to stress levels, future parent training programs should take into account the reality of parents of children with ASD, who are significantly more stressed than parents of children with typical development or with other neurodevelopmental disabilities [72, 73]. Effective parent training programs for toddlers with ASD should therefore limit parents' workload. The high level of stress in families of children with ASD may also explain why, even though parent training is associated with a decrease in stress and an increase in the sense of competence of parents of preschoolers with attention deficit hyperactivity disorder [74], the effects of such interventions are different for parents of children with ASD. Future studies should include an assessment of the effects of parent training interventions on parental stress and other well-being outcomes such as self-competence and empowerment.

Overall, the results of this review are consistent with those of a meta-analytic review of parent training programs for toddlers with behavior problems but without developmental disabilities [26]. The meta-analysis found greater effect sizes for parental outcomes than for children's outcomes. In fact, considering that parent-mediated interventions are by definition multilevel and involve the transfer of an intervention from the trainer to the parent and then from the parent to the child [58], parents have a role as change agents for their child's development. The assumption that parental outcomes are more direct than child outcomes explains the greater and more consistent effects on parental outcomes in Kaminski and colleagues' meta-analysis. Similarly, parent training interventions are likely to have greater effects on proximal parental outcomes such as knowledge about ASD, use of specific strategies, and attitudes than on more distal parental outcomes such as stress levels. Also, it is important to consider that parents need to first learn the different strategies; then it may take some time before they begin to implement the strategies in their everyday activities and in different settings [75], and still more time is needed before the effects on the children's behavior or skills are observable [76]. Consequently, effects of parent-mediated interventions may only become apparent weeks after the end of the intervention, giving time for changes to happen at the child level. To capture possible improvements after interventions, followup seems to be an important aspect of parent training study design that has been poorly assessed.

Finally, few studies looked specifically at parent-child interactions outcomes and most of them reported mixed results. The lack of scientific evidence prevents us from drawing definitive conclusions about the effects of parent training interventions on parent-child interactions. However, a review of parent-mediated interventions for children with ASD under 7 years of age reported strong and statistically significant positive changes in patterns of parent-child interactions [25]. Knowing that parent-mediated interventions are based on the assumption that the effects of the intervention depend on parents implementing the strategies in their interaction with their child and consequently on parents' responsiveness to their child [36], parent-child interactions are a very important outcome to assess. Future studies should include an assessment of parent training effects on parent-child interactions as a potential mediator of the effectiveness of parent-mediated interventions for toddlers with ASD.

## 5. Conclusion

This review shows that parent training interventions are a promising way to foster children's development by enhancing parent-child interactions. In fact, parents who received parent training interventions for their young child with ASD were able to learn and implement strategies to foster their child's development and were very satisfied with the program. However, poor evidence concerning children's skills, parents' well-being, and parent-child interactions outcomes prevents us reaching definitive conclusions regarding the effects of parent training interventions.

## Figures and Tables

**Table 1a:** (a)

Studies	Participant characteristics			Methods	Control condition (if applicable)
	*n* total (intervention group)	Age (months) mean (min–max)	Diagnosis		
Drew et al., 2002 [38]	24 (intervention = 12)	22.5	Childhood autism (ICD-10)	Randomized controlled trial	Local services: mixture of services
Mahoney and Perales, 2003 [36]	20	32.1	Autistic disorder or PDD	Preexperimental design: pre/postintervention	n/a
Mahoney and Perales, 2005 [37]	20	32.6	ASD	Preexperimental design: pre/postintervention	n/a
Wetherby and Woods, 2006 [39]	17	18.2 (12–24)	Provisional clinical diagnosis of ASD	Quasi-experimental design: pre/postintervention	Contrast group: no treatment
Rocha et al., 2007 [40]	3	31.7 (26–42)	Autistic disorder	Single subject multiple baseline across pairs of participants	n/a
Schertz and Odom, 2007 [41]	3	26.3 (22–33)	Strong early markers of ASD	Mixed methods: multiple-baseline design	n/a
Vismara et al., 2009 [34]	8	27.5 (10–36)	Autistic disorder (DSM-IV)	Case study: nonconcurrent multiple-baseline design	n/a
Gulsrud et al., 2010 [42]	34	30.6 (21–36)	ASD	Quasi-experimental design	n/a
Kasari et al., 2010 [43]	38 (intervention = 19)	30.8 (21–36)	Autistic disorder (DSM-IV)	Randomized waitlist control design	Waitlist: mixture of services
Oosterling et al., 2010 [44]	67 (intervention = 36)	32.9	Autistic disorder or PDD	Randomized controlled trial	Care-as-usual: daycare with speech and language therapy, motor therapy, music therapy, play therapy, and support for parents
Wong and Kwan, 2010 [45]	17 (intervention = 9)	26.5 (17–30)	ASD (DMS-IV)	Randomized waitlist control design	Waitlist: no intervention
Carter et al., 2011 [46]	55 (intervention = 29)	20.3 (15–25)	At risk of ASD	Randomized controlled trial	Control group: business as usual
Rogers et al., 2012 [10]	98 (intervention = 49)	21.0 (14–24)	At risk of ASD	Randomized controlled trial	Community group; mixture of services
Green et al., 2013 [47]	77 (intervention = 7)	8.4 (8–10)	Infant siblings of autistic probands	Case series with comparison groups; high risk (*n* = 37) and low risk (*n* = 33)	Comparison groups: no intervention
Steiner et al., 2013 [48]	3	12	Infant siblings of probands with ASD	Case series	n/a

**Table 1b:** (b)

Studies	Intervention characteristics				
	Type	Setting	Recommended frequency and duration	Hours of intervention	Children's targeted behaviors/abilities
Drew et al., 2002 [38]	Social-pragmatic joint attention-focused parent training	Home	3 h/6 weeks over 1 year	27 hours	Declarative acts combined with eye contact, nonverbal requests, object-function play, imitation of actions, and turn-taking.
Mahoney and Perales, 2003 [36]	Relationship-focused parent training	Clinic or home	1 h/week over 1 year	31 hours	Cognition, communication, socioemotional functioning, and motivation
Mahoney and Perales, 2005 [37]	Relationship-focused parent training (responsive teaching)	Clinic or home	1 h/week over 1 year	32.6 hours	Cognition, communication, socioemotional functioning, and motivation
Wetherby and Woods, 2006 [39]	Parent-child playgroup	Clinic	2x/week for 12 months	Not specified	Individualized social communication goals
	Naturalistic comprehensive parent training	Home	1x/week over 9 weeks	Not specified	
Rocha et al., 2007 [40]	Joint attention-focused parent-implemented intervention using natural behavior analytic strategies	Clinic	3x/week for 75 minutes over 6 weeks	At least 17 hours	Response of joint attention
Schertz and Odom, 2007 [41]	Joint attention-focused parent training	Home	1x/week for 9 to 26 weeks	Mean = 14 hours	Focus on faces, turn-taking, responding to joint attention, and initiating joint attention
Vismara et al., 2009 [34]	Comprehensive parent training incorporating behavioral and developmental strategies	Clinic	1 h/week over 12 weeks	12 hours	Attention and motivation, sensory social routines, dyadic engagement, nonverbal communication, imitation, joint attention, speech, and behavior
Gulsrud et al., 2010 [42]	Joint attention-focused caregiver training incorporating behavioral and developmental strategies	Clinic	3x/week for 30 minutes over 8 weeks	12 hours	Initiation and response of joint attention
Kasari et al., 2010 [43]	Joint attention-focused caregiver training incorporating behavioral and developmental strategies	Clinic	3x/week for 30 minutes over 8 weeks	12 hours	Initiation and response of joint attention
Oosterling et al., 2010 [44]	Social-pragmatic joint attention-focused parent training	Clinic Home	2 plenary sessions 2 h/week over 4 weeks + 3 h/6 weeks over 11 months	Not specified 32 hours	Declarative acts combined with eye contact, nonverbal requests, object-function play, imitation of actions, and turn-taking
Wong and Kwan, 2010 [45]	Communication-focused intervention with the child	Clinic	5x/week for 30 minutes for 2 weeks (10 sessions)	5 hours	Eye contact, gesture, and vocalization/words
	Communication-focused parent training				
Carter et al., 2011 [46]	Communication-focused parent group training	Clinic	1x/week over 8 weeks	Not specified	Two-way interaction, mature and conventional ways of communication
	Communication-focused parent training	Home	3x	Not specified	Communication for social purposes, understanding of language
Rogers et al., 2012 [10]	Comprehensive parent training incorporating behavioral and developmental strategies	Clinic	1x/week for 1 hour over 12 weeks	12 hours	Attention and motivation, sensory social routines, dyadic engagement, nonverbal communication, imitation, joint attention, speech, and behavior
Green et al., 2013 [47]	Developmental video-aided autism-specific parent training	Home	12 sessions of 1.5 hours over 5 months	18 hours	Social engagement and reciprocity
Steiner et al., 2013 [48]	Behavioral parent training	Clinic	8 sessions of 1 hour over 3 months	8 hours	Communication
		Home	2 sessions of 1 hour over 3 months	2 hours	

**Table 2a:** (a)

Studies	Time of followup after intervention assessment	Certainty of evidence	Child outcomes			
			Communication		Socioemotional functioning	Other
			Expressive	Receptive		
Drew et al., 2002 [38]	n/a	Preponderant	Mixed	Mixed	—	*Symptom severity*: none *Nonverbal IQ*: none
Mahoney and Perales, 2003 [36]	n/a	Suggestive	—	—	Mixed	—
Mahoney and Perales, 2005 [37]	n/a	Suggestive	—	—	Mixed	*Global development*: **positive**
Wetherby and Woods, 2006 [39]	n/a	Suggestive	Mixed	**Positive**	—	—
Rocha et al., 2007 [40]	3 months	Preponderant	—	PI: **positive** FU: mixed	—	—
Schertz and Odom, 2007 [41]	5 weeks	Suggestive	—	—	—	—
Vismara et al., 2009 [34]	Weekly over 4 weeks	Suggestive	PI: **positive** FU: **positive**	—	—	—
Gulsrud et al., 2010 [42]	n/a	Suggestive	—	—	Mixed	—
Kasari et al., 2010 [43]	n/a	Conclusive	—	—	—	*Play*: mixed
Oosterling et al., 2010 [44]	n/a	Preponderant	None	None	—	—
Wong and Kwan, 2010 [45]	n/a	Preponderant	Mixed		**Positive**	*Play*: mixed
Carter et al., 2011 [46]	4 months	Conclusive	PI: none FU: none	—	—	—
Rogers et al., 2012 [10]	n/a	Preponderant	None	None	—	*Adaptive behaviors*: none *Global development*: none *Symptom severity*: none
Green et al., 2013 [47]	n/a	Suggestive	Mixed	None	—	*Symptom severity*: none *Global development*: mixed *Visual attention*: mixed
Steiner et al., 2013 [48]	n/a	Suggestive	**Positive**	—	—	—

**Table 2b:** (b)

Studies	Parent outcomes				Parent-child interactions	
	Fidelity of implementation	Stress	Satisfaction	Other	Parent's engagement with child	Child's engagement with parent
Drew et al., 2002 [38]	—	None	—	—	—	—
Mahoney and Perales, 2003 [36]	—	—	—	—	Mixed	**Positive**
Mahoney and Perales, 2005 [37]	—	—	—	—	Mixed	**Positive**
Wetherby and Woods, 2006 [39]	—	—	—	—	—	—
Rocha et al., 2007 [40]	Clinic PI: **positive** Home PI: mixed	—	**Positive**	—	PI: **positive** FU: none	PI: mixed FU: mixed
Schertz and Odom, 2007 [41]	—	—	**Positive**	—	—	PI: mixed FU: mixed
Vismara et al., 2009 [34]	PI: **positive** FU: **positive**	—	—	—	—	PI: mixed FU: mixed
Gulsrud et al., 2010 [42]	—	—	—	—	**Positive**	—
Kasari et al., 2010 [43]	**Positive**	—	—	—	—	Mixed
Oosterling et al., 2010 [44]	—	—	—	—	None	None
Wong and Kwan, 2010 [45]	—	Mixed	—	—	—	—
Carter et al., 2011 [46]	—	—	**Positive**	—	PI: mixed FU: none	PI: none FU: none
Rogers et al., 2012 [10]	Mixed	—	—	*Therapeutic relationship*: **positive**	—	—
Green et al., 2013 [47]	—	—	—	*Social validity*: **positive**	Mixed	—
Steiner et al., 2013 [48]	**Positive**	—	**Positive**	—	—	—

PI:postintervention.

FU: followup.
